# Metagenomics Investigation on Baby Diaper Area Microbiome and Its Association with Skin pH and Dermatitis in the Diapered Area

**DOI:** 10.3390/microorganisms13112632

**Published:** 2025-11-20

**Authors:** Ping Hu, Andrew N. Carr, Mirjana Parlov, Dionne Swift, Jay P. Tiesman, Nivedita Ramji, Jennifer J. Schoch, Amber G. Teufel

**Affiliations:** 1 Corporate Functions R&D, The Procter & Gamble Company, Mason, OH 45040, USA; hu.p@pg.com (P.H.);; 2 Baby Care Life Sciences, The Procter & Gamble Company, Cincinnati, OH 45232, USA; carr.an@pg.com; 3 Department of Dermatology, University of Florida, Gainesville, FL 32611, USA

**Keywords:** baby, metagenomics, microbiome, diaper rash, skin, pH

## Abstract

Dermatitis in the diapered area (DDA) is the most common skin condition in infants and can cause significant pain and discomfort, leading to disturbed sleep, changes in temperament, and heightened concern and anxiety for caregivers. This study investigates the relationship between skin pH, microbiome composition, and DDA severity in 158 infants from China, the US, and Germany, focusing on the buttocks, perianal, and thigh regions. Significant variations in DNA biomass and microbiota profiles were noted. *Escherichia coli* and *Veillonella atypica* were linked to higher rash scores and elevated skin pH, while *Bifidobacterium longum* showed a negative correlation with buttocks pH and rash severity but not with perianal rash. Correlation patterns emerged for other species, like *Enterococcus faecalis*, between perianal and buttocks rashes. Functional analysis identified key categories, including lipid and fatty acid metabolism, cofactor, amino acid, and carbohydrate metabolism, homeostasis and osmolarity stress, and microbial virulence and oxidative stress response, which are vital for skin health, DDA, and pH regulation in infants. These findings underscore the importance of maintaining a mildly acidic skin pH and minimizing fecal and urine residues for optimal infant skin health, suggesting that microbiota significantly influence DDA development, and provide insights for future preventive strategies and therapeutic interventions.

## 1. Introduction

Infant skin is characterized by its soft and supple appearance, yet it is physiologically immature, delicate, and highly sensitive, requiring careful protection and support during the early years of life. Despite the critical role our skin plays in health and immunity from birth, there is sparce scientific literature on skin development, and even less is known about the contribution of the resident microbes. There is evidence that the infant skin microbiome may be impacted by the route of delivery, the retention of vernix, and caregivers’ habits and practices, including bathing frequency and skin care routines, which differ across the globe [1,2]. Dermatitis in the diapered area (DDA), commonly known as diaper rash or nappy rash, is the most prevalent inflammatory skin condition affecting non-toilet trained babies, with nearly every infant experiencing DDA at some point in their development [1,3,4,5]. This cutaneous inflammation arises from damage to the outermost layer of the epidermis, the stratum corneum, which is composed of corneocytes embedded in a lipid- and protein-rich matrix. Once this barrier is damaged, the breach allows for the entry of irritants from urine and feces to penetrate the skin and initiate an immune response visible as erythema (redness), papules and/or pustules. This condition not only causes discomfort for infants but also leads to sleep disturbances, increased crying and irritability, and significantly affects caregiver well-being contributing to parental stress [3,6,7]. The causes of DDA are multifactorial and include disrupted skin ultrastructure due to prolonged exposure to moisture and skin-on-skin rubbing (intertrigo), which exacerbates frictional damage to skin. Additionally, microbial factors play a crucial role in its development, as the skin’s natural microbiota can become disrupted, leading to the opportunistic overgrowth of harmful bacteria or yeast in warm, moist environments [8]. Skin contact with urine and stool are key drivers of rash, with the stool containing digestive enzymes (lipases and proteases) from the gut degrading and damaging skin. Prolonged exposure of skin to urine increases the risk of overhydration. Bacteria on the skin (either resident or from feces) contain enzymes (ureases) that convert the urea in urine to ammonia (pH = 11) that can increase skin pH, as well as activate several pH-dependent lipases and proteases that can damage skin lipids and protein, respectively. The elevation in skin pH can contribute to dysbiosis but also impairs normal skin function (e.g., desquamation) and slows skin healing. Some risk factors for DDA include prematurity, overhydrated skin, infrequent diaper changes, elevated skin pH, antibiotic use, and changes in the irritancy potential and frequency of stool (often diet related). Infants with certain medical conditions or those experiencing diarrhea are at higher risk [1]. To prevent DDA, caregivers are instructed to use high-quality, absorbent diapers, conduct frequent diaper changes, apply topical products, and clean the skin with water or a low pH baby wipe.

Skin pH is emerging as a crucial marker of skin health. The physiological importance of maintaining an acidic superficial skin pH is evidenced by the existence of four unique pathways that functionally provide redundancy to ensure skin acidity. These include the following: (1) generation of free fatty acids by the hydrolysis of phospholipids by secretory phospholipases (sPLA2); (2) sodium–hydrogen antiporter (NHE1); (3) catabolism of filaggrin (FLG) to acidic components of the natural moisturizing factor (NMF); and (4) release of protons during melanin transfer from melanocytes to keratinocytes [9]. The establishment of skin’s acidic nature begins at day 1, with near neutral skin pH at birth rapidly declining to values ranging from 4.5 to 5.5 within 2–3 weeks [10], which coincides with the initial establishment of the skin’s microbiome. Thus, the connection between skin pH and microbiome are inherent from the very beginning of life. This low pH supports the skin barrier and inhibits the growth of pathogenic microorganisms [11]. Lower skin pH levels are associated with reduced severity of DDA, highlighting the potential for pH management as a preventive strategy against DDA [12,13]. Moreover, skin pH can vary significantly across different populations due to factors such as diet, environmental conditions, and genetic predisposition.

Recent studies have revealed differences in skin pH among babies from diverse geographical backgrounds, including those from China, Germany, and the United States [1]. Babies in certain regions may exhibit higher skin pH levels, which coincides with an increased risk of DDA. Understanding these variations is essential for developing targeted interventions and skincare products that address the specific needs of infants in different cultural contexts. A balanced microbiome can help prevent the overgrowth of pathogenic bacteria and fungi, thereby reducing the risk of inflammation and irritation. The skin microbiome in the diapered area of infants, while sharing some structural similarities with adult skin communities, is heavily influenced by the persistent presence of moisture and fecal material in the occluded environment [14]. Unlike the adult skin microbiome, which is dominated by *Actinobacteria* (e.g., *Cutibacterium* and *Corynebacterium*), *Firmicutes* (e.g., *Staphylococcus*, *Streptococcus*, and *Lactobacillus*), *Proteobacteria*, and *Bacteroidetes*, the infant diaper area microbiome is heavily influenced by gut-derived bacteria such as *Bifidobacteria*, *Bacteroides*, *Enterobacteria*, *Eubacteria*, *Clostridium*, and *Lactobacillus* [14,15]. This microbiome is highly heterogenous across four anatomical sites: genitals, intertriginous folds, buttocks, and perianal region, with buttocks samples often showing higher levels of skin-associated bacteria, while perianal samples are enriched with gut-associated species like *Veillonella* and *Enterococcus* [16]. Typical microbial communities in the diaper area include purported beneficial bacteria such as *Bifidobacterium* and *Lactobacillus*, which may help maintain the skin’s protective barrier and support immune function [16]. Conversely, pathogens like *Staphylococcus aureus* and *Candida albicans* can proliferate under disrupted alkaline conditions [17], contributing to DDA [16]. Additionally, increased levels of *Finegoldia* and fecal coliforms have been associated with DDA. The interplay between the skin and gut microbiomes is especially important for infants, whose skin is more susceptible to environmental stressors and microbial imbalances, especially in the diaper area, which might impact the development of DDA. The skin–microbiome interaction should be considered bidirectional as it pertains to skin pH. Skin pH can play a crucial role in shaping the microbial environment on the skin, with lower (more acidic) pH generally promoting beneficial bacteria while inhibiting microbes considered to be pathogenic [18]. Conversely, elevated skin pH can disrupt this balance, allowing harmful microorganisms to thrive and potentially worsen conditions like diaper rash. At the same time, microbes can produce chemicals that influence their surrounding environment and self-select for optimal growth conditions. By managing skin pH with appropriate skincare products, caregivers can enhance microbial balance in the diaper area, potentially reducing the incidence and severity of diaper rash in infants. Despite the growing research on the interplay between skin pH, microbiome composition, and DDA severity, significant knowledge gaps remain. There is a critical need for comprehensive studies that directly examine how variations in skin pH affect the composition and function of the microbiome in the diaper area.

In our previous study involving 1791 infants [1], DDA was found to be most severe in Germany, followed by the USA, with the lowest rates in China. Chinese infants exhibited lower skin pH and improved skin barrier integrity, as indicated by reduced trans-epidermal water loss (TEWL). Additionally, Chinese caregivers reported more frequent diaper changes, higher use of topical products, more thorough cleaning routines after stooling, and less time in the overnight diaper, all of which may have contributed to lower DDA rates. The study also identified that higher humidity in the diaper area increased the risk of skin overhydration. The perianal region had the highest prevalence and severity of DDA, followed by the intertriginous (leg folds), genital, and buttock regions. These findings highlight the significance of geographic and body site differences in infant skin health, many of which are closely related to skin microbiome composition.

To deepen our understanding of the relationship between the skin microbiome and skin wellness in infants, we investigated a subset of 158 infants, focusing on three specific body sites: buttocks, perianal, and thighs. Our primary objective was to explore how microbial communities may influence or respond to the severity of diaper rash and inform better infant skincare practices. Specifically, we aimed to examine the relationships between skin pH, diaper rash severity, and microbiome composition across different geographical backgrounds. This study employs metagenomic analysis and statistical methods to thoroughly assess microbiome composition and its correlations with skin pH and rash severity. Previous research has indicated notable differences in microbial community composition at diaper sites, especially during instances of DDA, but these studies often relied on 16S rRNA sequencing, which limits species-level resolution and did not consider babies from different geographical locations. Thus, our study seeks to address these gaps using advanced technologies.

## 2. Materials and Methods

### 2.1. Study Design, Subject Recruitment, and Sample Collection

The samples analyzed were collected as part of a multinational study which recruited babies at locations in Beijing China, Hamburg Germany, and Cincinnati Ohio, USA, as previously described [1]. All procedures were conducted according to Good Clinical Practices and IRB/Ethics Board approval and informed consent (in the native language) was obtained in each country. Briefly, this was a cross-sectional study of 1791 babies (~600 from each country) recruited at each clinical site. Based on regional toilet-training habits, exclusively diaper-wearing infants were recruited between ages 2–8 months in China and 2–18 months in the USA and Germany. Skin pH, trans-epidermal water loss (TEWL), and relative humidity (RH) in the diapered region were measured as previously described [1]. The diaper rash severity was graded in both the buttocks and perianal regions using a publicly available, validated DDA scoring tool (7-point scale), as previously described [1]. Caregiver habits were collected via a questionnaire, including information on hygienic practices.

From the 1791 infants, D-Squame tape strips (D-100, CuDerm, Dallas, TX, USA) were collected from a subset of 300 infants across the 3 geographies: 100 each from China, Germany, and the U.S., covering the thigh, perianal, and buttock regions (Total 900 samples for DNA purification). Only 158 infants had sufficient microbial DNA for analysis from both the buttocks and perianal sites. Additionally, thigh microbiome samples from 70 infants (out of these 158 infants) contained enough DNA for sequencing and passed quality control.

### 2.2. Sample Preparation for Microbial Sequencing

Skin samples were collected using D-Squame tape strips at the thigh, buttocks, and perianal region. Thigh samples were collected approximately halfway between the hip and knee area. Perianal samples were collected in the perianal groove and buttock samples were collected on the same side as the perianal samples, on the lower buttocks (slightly below the center line to avoid overlapping TEWL measurement sites). Once applied, pressure was applied by a gloved hand and 3 circular motions were made before removing the tape with forceps. The first out of four collected strips was used for DNA extraction. DNA was extracted from tape strips using a modified Agencourt DNAdvance DNA Isolation Protocol Kit (A48706, Beckman Coulter Life Sciences, Indianapolis, IN, USA). The DNA extraction method is described in detail in the Supplementary Information. It consisted of bead beating, protein digestion, and DNA purification with magnetic beads. The extractions were performed manually in a 96-well plate for the first part of the protocol, then the magnetic beads addition, ethanol washes, and elution steps were performed using the Beckman FX robot (Beckman Coulter Life Sciences, Indianapolis, IN, USA). The sample locations in the 96-well isolation plates were randomized to minimize potential biases.

Extracted DNA was quantified using the Thermo Scientific Varioskan LUX Multimode Microplate Reader (Thermo Fisher, Hanover Park, IL, USA) and dsDNA HS (High Sensitivity) Assay Kit (Q3323, Thermo Fisher, Hanover Park, IL, USA). Then, DNA was normalized to 0.2 ng/μL and processed using Illumina’s Nextera XT DNA Library Preparation Kit (FC-131-1096, Illumina, San Diego, CA, USA) per the manufacturer’s instructions. The library DNA was purified and size selection was conducted with 0.6× Agencourt AMPure XP beads (A63881, Beckman Coulter Life Sciences, Indianapolis, IN, USA) [19]. The resulting DNA was quantified on the Qubit using the dsDNA HS kit and run on an Agilent Bioanalyzer with the High Sensitivity DNA Kit (5067-4626, Agilent Technologies, Santa Clara, CA, USA), to confirm that the size of the DNA ranged from 250 to 1000 bp. The libraries were normalized to 2 nM and equal volumes were pooled. PhiX DNA (FC-110-3002, Illumina, San Diego, CA, USA) was added at 1% to serve as an internal sequencing control. These libraries were then sequenced on high-output flow cells (2 × 150 paired-end reads) on a NextSeq 500 instrument (Illumina, San Diego, CA, USA).

### 2.3. Bioinformatics Analysis

Sequence bases with quality scores below 30 were discarded from both 3′ and 5′ end using cutadapt version 4.0. The remaining reads were further filtered by samtools version 1.21 [20], bowtie2 version 2.5.1 [21], and SNAP version 2.0.3 [22] to remove reads mapped to the human genome (GRCh38) and Phix genome. Read pairs with a read mean quality score below 30 or shorter than 75% of the read length (i.e., 105 bp) were also discarded. FastQC v0.12.1 [23] was performed on the remaining reads (Pass Filter Reads). Pair-end FASTQ files were separately merged in case multiple read files were used for the same sample. Samples with read numbers less than the negative controls were discarded. Analysis of the taxonomic distribution was performed by MetaPhlAn4.06 [24], a profiling algorithm using clade-specific marker genes and Kraken2 version 2.1.3 [25], a K-mer-based algorithm with an expanded database including ~3000 fungal genomes from NCBI. Gene Ontology and biological pathways were assessed using HUMAnN3.8 [26]. There were 152 subjects with matching microbiome metagenomics samples from both perianal and buttocks sites. Diversity measurements including alpha diversity (observed species and Shannon diversity) and beta diversity were performed with R Vegan package version 2.7.1 [27].

### 2.4. Statistical Analysis

All data analyses were performed using R software (version 4.1.3, vegan 2.7.1, phyllode 1.38.0). Paired *t*-tests were used to test meta data differences among different anatomical sites when there were the same number of subjects involved. Non-paired *t*-tests were used when comparing different geographic locations or when comparing a non-equal number of subjects between different groups. Differences in the microbial community between groups were assessed using the Wilcoxon rank test for two-group testing and the Kruskal–Wallis’s test for multi-group analysis. Nonmetric multidimensional scaling (MDS) analysis was performed to show community clustering of samples using Bray–Curtis’s similarities or the Jaccard similarity, which served to evaluate beta diversity. Pairwise Wilcoxon Rank Sum tests and Adonis tests were performed to test microbial differences among different populations to establish the *p* values. A false discovery rate (FDR) correction was applied to adjust the significance level and differences were considered significant at *p* ≤ 0.05. Complex Heatmap was used to generate correlation and a heatmap [28].

## 3. Results

### 3.1. Geographic and Site Variations in Baby Skin Microbiome

#### Biomass (DNA Amount) from Infant Skin Tape Strips

**Figure 1:**
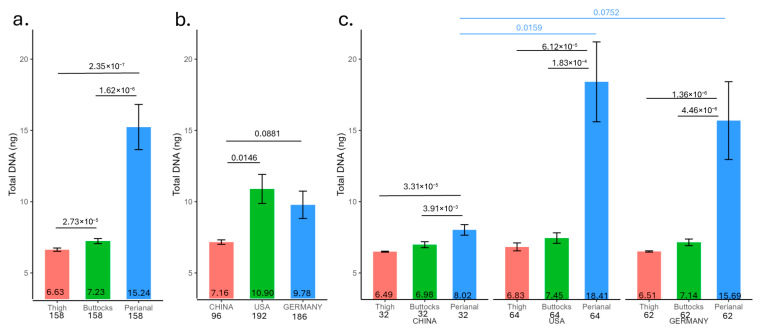
Average purified total DNA yield (ng) from 158 infant skin tape strips (158 buttocks, 158 perianal, and 158 thigh samples). (**a**) By anatomical site, (**b**) by geographic location, and (**c**) by site and geographic location. The average is displayed at the bottom of the bar while the number of samples per site is labeled on the *x*-axis. *t*-test *p* values were displayed if equal to or less than 0.1.

DNA was purified from the tape strips of 158 infants (32 from China, 62 from Germany, and 64 from the US) for microbiome analysis (Table S1). Significant variations in DNA quantities were observed across different anatomical sites (Figure 1a). The lowest DNA concentration was found in the thigh (6.63 ng), followed by the buttocks (7.23 ng), and the perianal region, which exhibited the highest mass (15.24 ng). Moreover, samples from Chinese infants contained less DNA overall compared to those from the US and Germany (Figure 1b, China: 7.16 ng, US: 10.90 ng, and Germany: 9.78 ng), particularly in the perianal region (Figure 1c, China perianal: 8.02 ng; US perianal: 18.41 ng; and Germany perianal: 15.69 ng). US and Germany DNA amounts showed no significant differences. The DNA amount showed a pattern consistent with the occurrence and severity of rashes. Notably, only 70 infants had sufficient DNA purified from the thigh for metagenomic sequencing.

### 3.2. α-Diversity Measurement from Infant Skin Tape Strips

#### 3.2.1. Observed Microbial Species from Infant Skin Tape Strips

**Figure 2:**
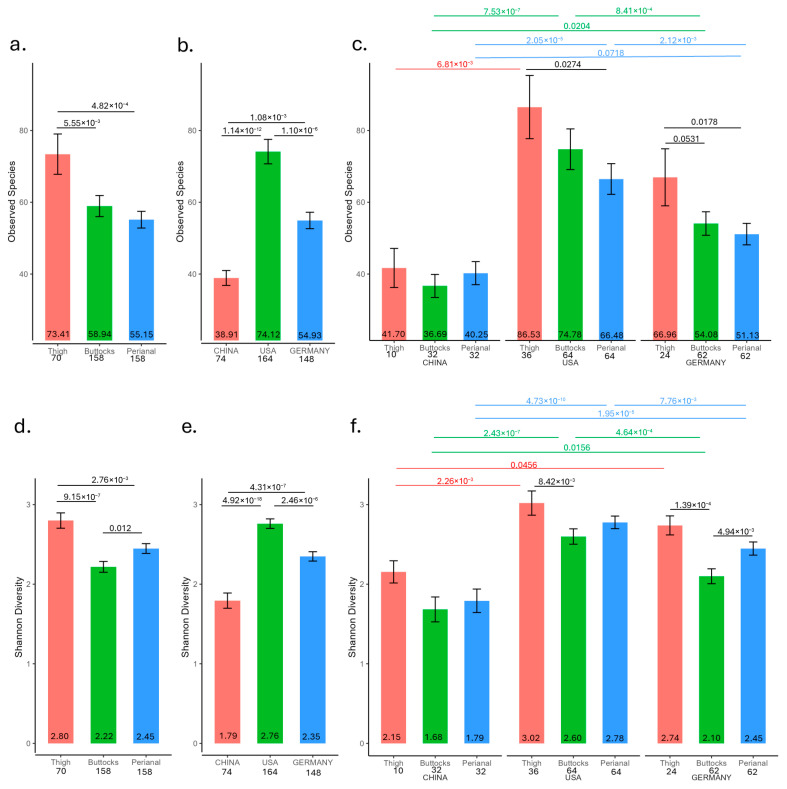
α-Diversity measurements from 158 infant skin tape strips (158 buttocks, 158 perianal, and 70 thigh samples). (**a**) Observed microbial species by anatomical site, (**b**) observed microbial species by geographic location, (**c**) observed microbial species by site and country. (**d**) Shannon Diversity Index by anatomical site, (**e**) Shannon Diversity Index by geographic location, and (**f**) Shannon Diversity Index by site and country. The average is displayed at the bottom of the bar while the number of samples per site is labeled on the *x*-axis. *t*-test *p* values were displayed if equal to or less than 0.1.

The highest number of microbial species was observed on the thigh (73), followed by the buttocks (59), while the perianal samples had the lowest count (55) (Figure 2a, Table S2). Notably, there were no significant differences between the buttocks and perianal samples. Regarding geographic differences, Chinese samples had the fewest microbial species (39), followed by German samples (55), and US samples (74) (Figure 2b). This pattern persisted across different anatomical sites (Figure 2a,c).

#### 3.2.2. Shannon Diversity from Infant Skin Tape Strips

The Shannon diversity measurement revealed significant differences across body sites, with the highest diversity observed in the thigh and the lowest in the buttocks (Figure 2d). Geographic location also influenced diversity, with US samples showing the highest Shannon diversity and Chinese samples the lowest (Figure 2e). Notably, significant differences were found between buttocks and perianal samples across geographic locations (Figure 2f). However, the only significant difference among thigh samples was that Chinese samples had lower Shannon diversity compared to US samples (Figure 2f). In German samples, the thigh exhibited significantly higher Shannon diversity than both the buttocks and perianal samples. In US samples, the thigh also showed significantly higher Shannon diversity compared to the buttocks. However, there were no significant differences between buttocks and perianal samples across any geographic site.

### 3.3. Infant Skin pH and Diaper Rash Severity in the Population with Metagenomics Data

Within this cohort, skin pH measurements from different body sites were significantly different with the average buttocks’ pH 5.44, average genital pH 5.62, and both were elevated compared to the average thigh pH 5.22. Perianal rash was generally more severe than the buttocks (paired *t*-test *p* = 3.77 × 10^−31^), with an average perianal rash score of 1.00 and an average buttocks rash score of 0.22.

**Figure 3:**
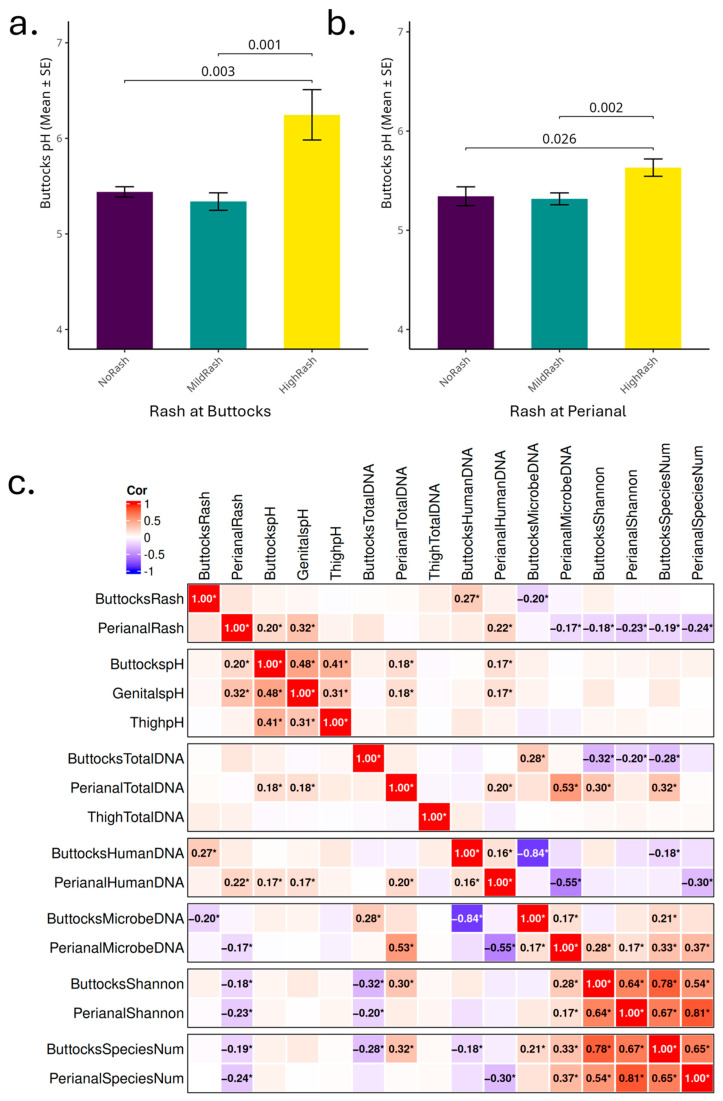
Correlation between infant skin pH, rash score, and microbial measurements (DNA, species number, and Shannon diversity). (**a**) Buttocks skin pH differs across no rash (NoRash, rash score = 0), mild rash (MildRash, rash score 0.5–1), and high rash (HighRash, rash score ≥ 1.5) groups. Pairwise *t*-test *p* value with *p* ≤ 0.05 are displayed. (**b**) Perianal skin pH differs across no rash (NoRash, rash score = 0), mild rash (MildRash, rash score 0.5–1), and high rash (HighRash, rash score ≥ 1.5) groups. Pairwise *t*-test *p* value with *p* ≤ 0.05 are displayed. (**c**) Spearman correlation plot showing relationships among infant skin pH, rash score, total DNA, microbial DNA, human DNA, Shannon diversity, and observed microbial species number. Positive correlations are shown in red, negative in blue, with color intensity reflecting the correlation coefficient (−1 to 1). Only correlations with *p* ≤ 0.05 are displayed with *.

Infants with higher buttocks diaper rash scores (≥1.5) had significantly elevated buttocks skin pH levels (average buttocks pH = 6.25) compared to those without rash (score = 0; average buttocks pH = 5.44, *p* = 0.003) and those with mild rash scores (0 < score ≤ 1; average buttocks pH = 5.34, *p* = 0.001) (Figure 3a). Similarly to the findings for the buttocks, infants with high perianal rash scores had significantly higher buttocks skin pH (average pH = 5.63) compared to those without perianal rash (average pH = 5.34, *p* = 0.026) or with mild perianal rash (average pH = 5.32, *p* = 0.002) (Figure 3b). No significant differences in skin pH were found between the no rash and mild rash groups. Infants with a buttocks pH greater than 5.5 showed higher buttocks diaper rash prevalence (average buttocks rash score = 0.29) compared to those with a pH ≤ 5.5 (average buttocks rash score = 0.16, *p* = 0.05). Given the proximity of the pH measures near the perianal and buttock regions, we also compared perianal rash scores and buttock pH. Infants with buttocks pH > 5.5 had significantly higher perianal rash scores (average perianal rash score = 1.13) than those with a buttocks pH ≤ 5.5 (average perianal rash score = 0.89, *p* = 0.015). Significant differences were not observed for trans-epidermal water loss (TEWL), front and back temperature, and relative humidity (RH) when the samples were categorized into high, low, and mild rash groups in this cohort.

Spearman correlation analysis (Figure 3c) indicated that perianal diaper rash positively correlated with both buttocks skin pH, genital skin pH, and perianal human DNA, and negatively correlated with perianal microbial DNA, Shannon diversity, and observed microbial species number from both the perianal and buttocks. In both the buttocks and perianal regions, rash scores showed a positive correlation with human DNA and a negative correlation with microbial DNA obtained from the tape strips. This may be attributed to rash-associated tissue damage and easier removal of cells by the collection tape. While most directional correlations are consistent between the buttocks and perianal sites, total DNA showed different patterns: perianal total DNA positively correlated with both buttocks Shannon diversity and buttocks observed microbial species, while buttocks total DNA showed negative correlation with these α-diversity measurements. The positive correlation of perianal total DNA with skin pH is also indicated when skin pH is higher as more DNA will be collected on the tape strips from the perianal site.

Though thigh skin pH correlated well with both buttocks and perianal skin pH, it was not associated significantly with any of the other measurements listed here. Thigh total DNA also did not show any significant association with other measurements in this analysis. As the thigh is the site outside of the diaper area and normally free of diaper rash, it was omitted from the subsequent analysis.

### 3.4. Infant Diaper Area Microbiota Composition and Association with Skin pH and Diaper Rash Severity

#### 3.4.1. Infant Skin Microbiome Compositional Differences by Geographical Location

**Figure 4:**
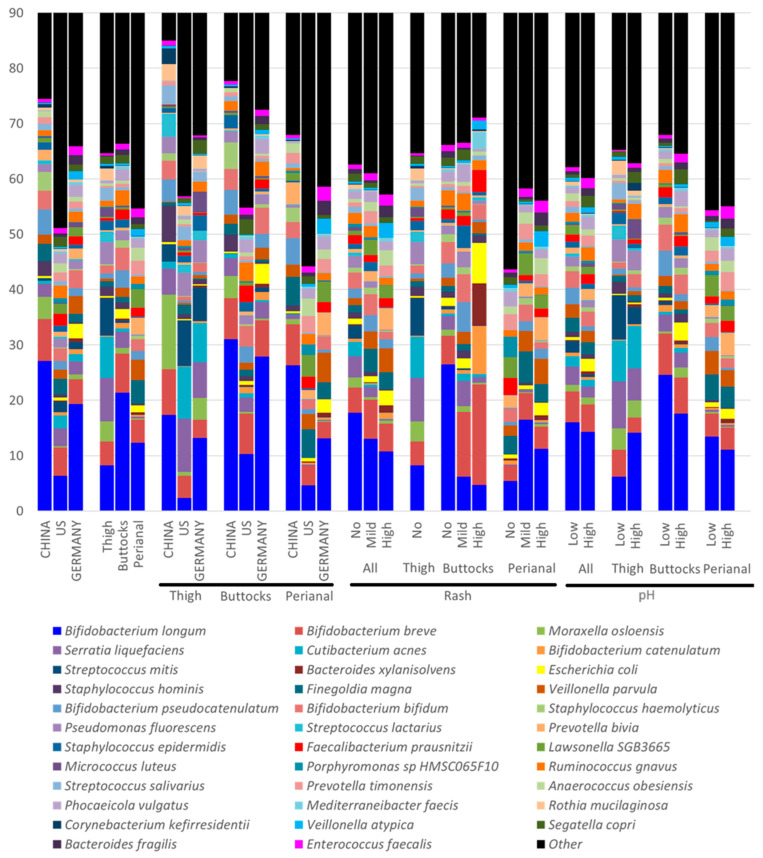
Composition of the infant skin microbiome from tape strips by anatomical sites, geographic locations, rash severity, and skin pH. Microbial species with a relative abundance of 2.5% or greater in any category were included.

The composition of the baby skin microbiome varied significantly across three geographic regions (Figure 4, Table S3). For infants in China, the top five species were *Bifidobacterium longum* (27.14%), *Bifidobacterium breve* (7.52%), *Bifidobacterium pseudocatenulatum* (4.54%), *Moraxella osloensis* (3.98%), and *Staphylococcus haemolyticus* (3.42%). For infants in Germany, the top five were *B. longum* (19.33%), *B. breve* (4.51%), *Veillonella parvula* (3.24%), *Bifidobacterium bifidum* (3.23%), and *Escherichia coli* (2.57%). For infants in the U.S, the top five species included *B. longum* (6.35%), *B. breve* (5.11%), *Serratia liquefaciens* (3.21%), *Finegoldia magna* (2.85%), and *Cutibacterium acnes* (2.38%). *E. coli* had the highest abundance in Germany samples (China 0.36%, US 0.58%).

#### 3.4.2. Infant Skin Microbiome Compositional Differences by Anatomical Sites

Significant microbial differences were observed among the three anatomical sites (Figure 4, Table S4). For the buttocks, the top five species were *Bifidobacterium longum* (21.41%), *Bifidobacterium breve* (7.00%), *Bifidobacterium bifidum* (4.13%), *Bifidobacterium pseudocatenulatum* (3.08%), and *Sphingomonas liquefaciens* (2.86%). In the perianal area, the leading species were *B. longum* (12.35%), *Finegoldia magna* (4.29%), *B. breve* (4.11%), *Veillonella parvula* (3.66%), and *Lawsonella SGB3665* (3.33%). For the thigh, the top five species included *B. longum* (8.22%), *S. liquefaciens* (7.84%), *Cutibacterium acnes* (7.44%), *Streptococcus mitis* (6.76%), and *B. breve* (4.37%). The thigh microbiome differed significantly from the buttocks and perianal microbiomes, with a greater variety of bacteria identified from human skin samples, while the perianal samples predominantly contained gut–fecal bacteria. For example, *C. acnes* was most abundant in thigh samples (7.44%) and least in perianal samples (0.04%). Similarly, species commonly found in the human skin microbiome, such as *Staphylococcus epidermidis* (thigh 1.75%, buttocks 1.44%, and perianal 0.24%) and *Micrococcus luteus* (thigh 1.55%, buttocks 0.21%, and perianal 0.03%), exhibit the highest abundance in thigh samples, followed by the buttocks, with very low levels in perianal samples.

The most-detected species are bacteria; however, fungal species such as *Malassezia globosa* (thigh 0.04%, buttocks 0.03%, and perianal 0%), *Malassezia restricta* (thigh 0.10%, buttocks 0.001%, and perianal 0%), and *Candida albicans* (thigh 0%, buttocks 0.001%, and perianal 0%) were found in much lower amounts (Table S4).

#### 3.4.3. Infant Skin Microbial Differences by Rash Severity

In the buttocks samples, 27 bacterial species exhibited Spearman correlations with a *p*-value of ≤0.05 in relation to buttocks rash scores and four species had a relative abundance of at least 1% in any of the groups categorized as no rash (sash score = 0; *n*= 119), mild rash (rash score = 0.5 or 1; *n* = 34), and high rash (rash score > 1; *n* = 5) (Table S5). Notably, *Bifidobacterium longum* showed a negative correlation with rash severity (*p* = 0.0002). In contrast, *Bifidobacterium catenulatum*, *Staphylococcus epidermidis*, and *Finegoldia magna* were positively correlated with rash scores. However, their relative abundances did not follow a consistent pattern of high rash > mild rash > no rash; specifically, *S. epidermidis* and *F. magna* had higher relative abundances in mild rash samples compared to both the no rash and high rash groups.

In the perianal samples, 116 bacterial species exhibited Spearman correlations with a *p*-value of ≤0.05 related to perianal rash scores. Twelve species had a relative abundance of at least 1% in the groups categorized as no rash (rash score = 0; *n* = 30), mild rash (rash score = 0.5 or 1; *n* = 67), and high rash (rash score > 1; *n* = 61) (Table S6). *Veillonella atypica*, *Veillonella parvula*, *Enterococcus faecalis*, *Phocaeicola dorei*, and *Veillonella seminalis* were positively correlated with perianal rash scores. Conversely, *GGB4277 SGB5832*, *Faecalibacterium prausnitzii*, *Sergatella copri*, *Phocaeicola vulgatus*, *Porphyromonas* sp. *HMSC065F10*, *Veillonella ratti*, and *Bacteroides caccae* showed negative correlations with perianal rash scores.

#### 3.4.4. Infant Skin Microbial Differences by Skin pH

In the buttocks samples, 47 microbial species exhibited Spearman correlations with a *p*-value of ≤0.05 in relation to buttocks skin pH measurements. Among these, only two species—*Escherichia coli* and *Moraxella osloensis*—had a relative abundance greater than 1% in either the high pH group (pH > 5.5; *n* = 72) or the low pH group (pH ≤ 5.5; *n* = 86) (Table S7). Notably, *Staphylococcus aureus* and *Malassezia restricta*, associated with atopic dermatitis and seborrheic dermatitis, also showed a positive correlation in the buttocks area. Our previous study [16] indicated a positive association of *E. coli* and *S. aureus* with diaper rash.

In the perianal samples, 37 microbial species demonstrated Spearman correlations with a *p*-value of ≤0.05 concerning skin pH measurements. Four species—*E. coli*, *Prevotella bivia*, *Sergatella copri*, and *Veillonella seminalis*—had a relative abundance greater than 1% in either the high or low pH groups (Table S8).

#### 3.4.5. Selected Microbial Species and Their Associations with Rash Severity and Skin pH

**Figure 5:**
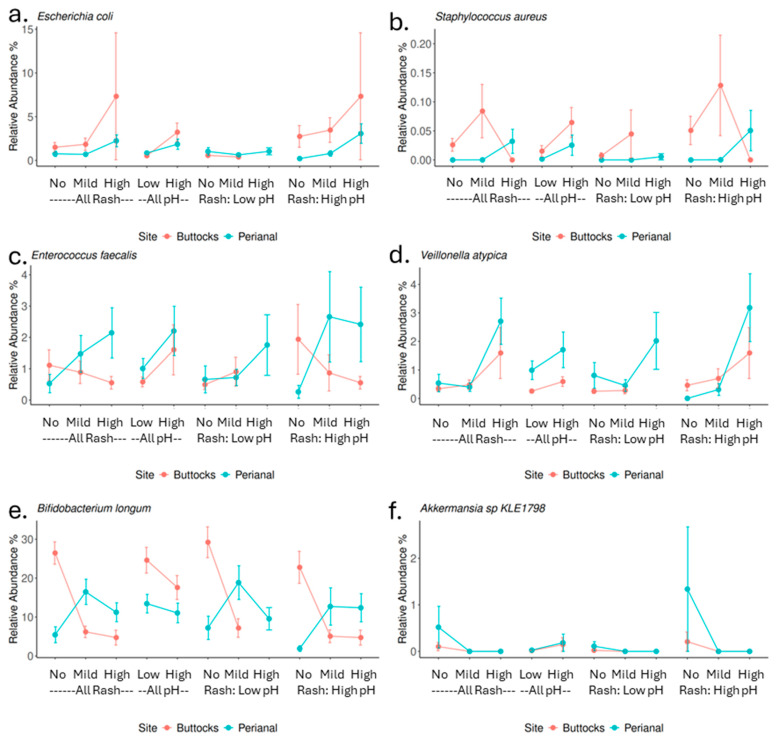
Selected microbial species and their associations with rash and pH in diapered areas (perianal and buttocks). The blue line indicates the relative abundance in perianal samples, while the red line represents the relative abundance in buttocks samples. The species depicted are as follows: (**a**) *Escherichia coli*, (**b**) *Staphylococcus aureus*, (**c**) *Enterococcus faecalis*, (**d**) *Veillonella atypica*, (**e**) *Bifidobacterium longum*, and (**f**) *Akkermansia* sp. *KLE1798*.

Several microbial species were examined further for their associations with skin pH and the severity of diaper rash (Figure 5). Our previous work [16] indicated that diaper rash is linked to increased levels of fecal coliforms, such as *Enterococcus*, and a lower percentage of *Staphylococcus*. Conversely, higher levels of *Staphylococcus aureus* were observed in high rash samples.

In this study, *Escherichia coli*, a prevalent fecal coliform in both perianal and buttocks samples, showed a positive association with skin pH and rash severity in both areas (Figure 5a). Although present in lower abundance, *S. aureus* was significantly higher in high pH and high in perianal rash samples (Figure 5b). *Enterococcus faecalis*, another common fecal coliform, was enriched in both high rash and high pH perianal samples (Figure 5c). Several *Veillonella* species were notably enriched in the perianal region, particularly associated with perianal rash. For instance, *Veillonella atypica* was significantly more abundant in high pH and high rash samples (Figure 5d).

*Bifidobacterium longum*, the most abundant species in this study, showed a negative correlation with both buttock pH and rash severity, being present in higher amounts in no rash buttocks samples. Interestingly, in perianal samples, it was found in greater quantities in high and mild rash samples compared to no rash samples (Figure 5e). Another noteworthy species is *Akkermansia* sp. *KLE1798*, which was rarely detected in other samples but was prevalent in no rash samples, particularly no perianal rash samples (Figure 5f).

### 3.5. Biological Functional Analysis of Infant Diaper Area Microbiota and Their Association with Skin pH and Diaper Rash Severity

#### 3.5.1. Relative Abundance of Infant Diaper Area Microbiota Biological Pathways and Their Association with Skin pH and Diaper Rash Severity

A total of 182 pathways showed significant Spearman correlations (*p*-value ≤ 0.05) with pH or rash in either buttocks or perianal samples (Figure S1): 24 pathways correlated with buttocks rash, 111 pathways correlated with buttocks pH; in perianal samples, 82 pathways correlated with perianal rash and 61 pathways correlated with buttock pH.

**Figure 6:**
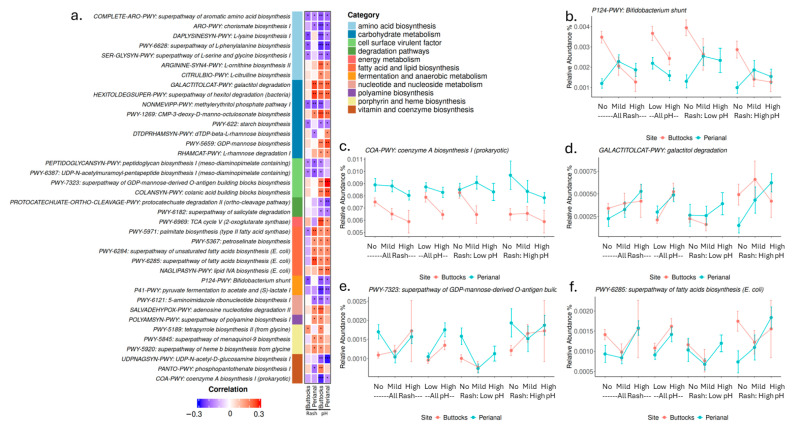
Microbial pathway correlated with rash and pH. (**a**) Heatmap of selected microbial pathways showing Spearman correlations with skin pH or rash scores (*p* ≤ 0.05) in perianal or buttocks sites. The side color bar indicated functional groups of these pathways. The panel shows Spearman correlation coefficients with pH or rash scores (*p* ≤ 0.05) in perianal or buttocks sites. (Significant level is indicated with *: *p* ≤ 0.05: *, FDR ≤ 0.1: **, FDR ≤ 0.05: ***). (**b**) Bifidobacterium shunt pathway, (**c**) Prokaryotic coenzyme A biosynthesis I (**d**) Galactitol degradation (**e**) Super pathway of GDP-mannose-derived O-antigen building block biosynthesis pathway. (**f**) Super pathway of fatty acids biosynthesis (*E. coli*).

These pathways can be categorized into several domains (Figure 6a), including amino acid biosynthesis, carbohydrate metabolism, cell surface virulent factors, degradation pathways, energy metabolism, fatty acid and lipid biosynthesis, fermentation and anaerobic metabolism, nucleotide and nucleoside metabolism, polyamine biosynthesis, porphyrin and heme biosynthesis, and vitamin and coenzyme biosynthesis. Selected examples, along with their relative abundances, Spearman correlation coefficients, and *p*-values, are displayed in Figure 6.

Like the profile of *Bifidobacterium longum* (Figure 5e), the Bifidobacterium shunt pathway (Figure 6b) showed a negative correlation with skin pH (in both perianal and buttocks sites) and buttock rash severity. However, in the perianal region, higher levels of the Bifidobacterium shunt pathway were associated with mild and high rash groups, particularly at lower skin pH levels. This pathway is crucial for fermenting carbohydrates, especially oligosaccharides, to produce short-chain fatty acids (SCFAs) and other metabolites. The distinct microbial profiles observed in the perianal region may indicate that signaling pathways activated during rash contribute to the initiation and acceleration of skin healing.

Several pathways related to amino acid biosynthesis and vitamin and cofactor biosynthesis showed negative correlations with either pH or rash severity, such as Prokaryotic coenzyme A biosynthesis I (Figure 6c). Carbohydrate metabolism is another major category closely associated with skin pH and rash severity. Notably, some pathways, such as Galactitol degradation (Figure 6d), GDP-mannose biosynthesis, and L-rhamnose degradation I, displayed positive associations with both pH and rash, while starch biosynthesis negatively correlated with both.

Mannose is critical for synthesizing glycoproteins and glycolipids, which are essential for various cellular functions, including cell recognition, signaling, and adhesion. The GDP-mannose biosynthetic pathway is vital for many bacteria, providing the necessary mannose residues for O-antigen synthesis. The O-antigen, a component of lipopolysaccharides (LPSs) found in the outer membrane of Gram-negative bacteria, plays a crucial role in bacterial virulence, immune evasion, and host interaction. Variations in O-antigen structures can help bacteria evade immune detection or promote adherence to host tissues. Interestingly, the super pathway of GDP-mannose-derived O-antigen building block biosynthesis (Figure 6e) was positively correlated with both pH and rash. In contrast, pathways associated with Gram-positive bacteria, such as peptidoglycan biosynthesis I, showed negative associations with both skin pH and rash severity. Several fatty acid biosynthesis pathways exhibited negative correlations with buttocks rash but positive correlations with skin pH and perianal rash, as seen in the super pathway of fatty acid biosynthesis (*E. coli*) (Figure 6f).

#### 3.5.2. Infant Diapered Area Microbiota Gene Functions: Gene Ontology Associations with Skin pH and Diaper Rash

**Figure 7:**
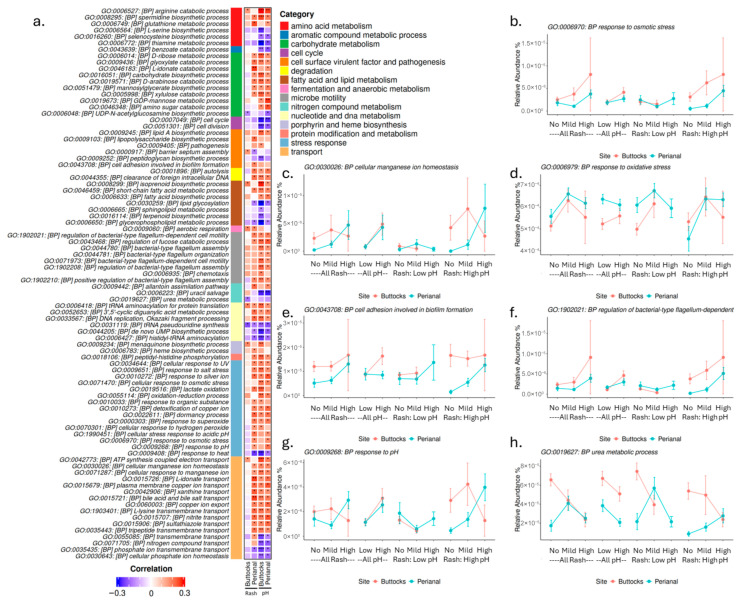
Microbial gene biological functions correlated with rash and pH. (**a**) Heatmap of selected microbial Gene Ontology (GO) terms showing Spearman correlations with skin pH or rash scores (*p* ≤ 0.05) in perianal and buttocks sites. The side color bar indicates the functional groups of these terms. Panel displays Spearman correlation coefficients with pH or rash scores (*p* ≤ 0.05) for both sites. (Significant level is labeled as: *p* ≤ 0.05: *, FDR ≤ 0.1: **, FDR ≤ 0.05: ***). The following functions are highlighted: (**b**) response to osmotic stress, (**c**) cellular manganese iron homeostasis, (**d**) response to oxidative stress, (**e**) cell adhesion involved in biofilm formation, (**f**) regulation of bacterial-type flagellum-dependent cell motility, (**g**) response to pH, and (**h**) urea metabolic process.

To better understand the relationship between microbiota gene functions and diaper rash severity, Gene Ontology (GO) terms were examined for their relative abundance and association with skin pH and diaper rash. This analysis is crucial because traditional pathway analyses often focus on metabolic pathways and may overlook specific gene functions. In total, 625 GO Biological Process (BP) terms exhibited significant Spearman correlations (*p* ≤ 0.05) with either skin pH or diaper rash severity. Specifically, for the buttock samples, 91 terms correlated with rash severity, while 355 terms correlated with skin pH. In the perianal samples, there were 265 terms associated with rash and 233 with pH. We selected a subset of GO terms with significant correlations across multiple comparisons, and their relative abundances, correlation coefficients, and *p*-values are presented in Figure 7 and Figure S2.

These GO terms can be categorized into several functional groups (Figure 7a), including (1) amino acid metabolism, (2) aromatic compound metabolic process, (3) carbohydrate metabolism, (4) cell cycle, (5) cell surface virulent factor and pathogenesis, (6) degradation, (7) fatty acid and lipid metabolism, (8) microbe motility, (9) fermentation and anaerobic metabolism, (10) nitrogen compound metabolism, (11) nucleotide and DNA metabolism, (12) porphyrin and heme biosynthesis, (13) protein modification and metabolism, (14) stress response, (15) transport, and (16) vitamin and cofactor metabolism.

One of the largest categories is stress response, where terms such as “Response to Osmotic Stress” (Figure 7b) correlated positively with both skin pH and rash severity in both buttocks and perianal samples. Similarly, responses to high salt and organic substances exhibited comparable patterns. Additionally, functions related to transport, such as cellular manganese ion homeostasis (Figure 7c), showed strong positive correlations with skin pH and perianal rash severity.

Increased oxidative stress may also be linked to higher skin pH and rash severity, as indicated by “Response to Oxidative Stress” (Figure 7d). Several GO terms associated with biofilm formation, virulence, and pathogenicity also showed positive correlations with skin pH and rash severity. For example, “Cell Adhesion Involved in Biofilm Formation” (Figure 7e) correlated positively with these factors. Furthermore, the regulation of bacterial-type flagellum-dependent cell motility (Figure 7f) demonstrated a positive correlation with skin pH and rash severity, particularly in high pH samples, where the abundance of motility genes increased in high rash samples.

The term “Response to pH” (Figure 7g) positively correlated with skin pH in both buttocks and perianal regions but exhibited different profiles for rash severity. In the perianal samples, the response increased with rash severity; however, in buttocks samples, it decreased with high rash severity in samples with high buttocks skin pH. These differences in microbial gene abundance between buttocks and perianal regions may be attributed to environmental factors, such as increased exposure to urine and fecal matter in the perianal area and may be a response to skin acidification pathways activated to drive down skin pH, increase cell proliferation and repair, and speed healing. Interestingly, the urea metabolism process (Figure 7h) showed a negative correlation with skin pH and buttocks rash severity, while in high pH perianal samples, it exhibited a positive correlation with perianal rash severity.

## 4. Discussion

A significant association between elevated buttocks skin pH levels and increased severity of DDA was found in this study, reflecting the skin microbiome and its functional changes, consistent with previous findings linking lower skin pH and skin health [1,13]. Specifically, infants with buttocks pH levels above 5.5 are at a heightened risk for developing DDA, underscoring the importance of monitoring skin pH as a routine aspect of infant care. Additionally, our results suggest that in the perianal region, factors such as osmolarity and cleanliness may be important factors influencing DDA in addition to maintaining a mildly acidic pH.

The total DNA yield from skin tape samples corresponds to the rash severity (Figure 1). The human DNA extracted from these tapes shows a positive correlation with rash severity, while the microbe DNA exhibits a negative correlation (Figure 3c). When the skin barrier is compromised due to rash conditions, the integrity of the stratum corneum is disrupted and cell turnover accelerated, facilitating easier detachment of skin cells by tape strips and increased exposure of smaller living cells. This might explain the resulting higher quantity of extracted human DNA for high-rash infants. Since all tape strips are of the same size and adhesive strength, this might also explain how a greater attachment of human cells could reduce the capacity for microbial cells to adhere to a greater contact area and cause deeper penetration of the microbes below the stratum corneum surface, leading to the observed strong negative correlation between microbial and human DNA yields. Elevated skin pH in the perianal region, accompanied by higher DNA yield, further underscores the relationship between pH and rash.

A diverse skin microbiome is widely recognized as essential for maintaining healthy skin. High microbial diversity supports skin barrier function, immune regulation, and protection against pathogens, while reduced diversity (dysbiosis) is linked to inflammatory skin conditions such as atopic dermatitis, acne, and psoriasis [29,30,31]. The observation that the thigh microbiome has the highest microbial diversity, followed by the buttocks and that the lowest diversity is in the perianal microbiome, agrees with this hypothesis (Figure 2a). Additionally, perianal microbial diversity is significantly higher in subjects without rashes compared to those with mild or severe rashes. (Figure S3).

While microbial diversity is important, microbial composition and function also play key roles in infant skin health. For example, Chinese infants exhibit lower microbial diversity than German and US infants (Figure 2b,e); however, German infants in this study have higher overall rash scores. A closer examination of the microbiota reveals that German samples have the greatest abundance of *E. coli* (China: 0.36%, Germany: 2.57%, and US: 0.58%), *Enterococcus faecalis* (China: 0.65%, Germany: 1.55%, and US: 0.98%), and *Veillonella atypica* (China: 0.29%, Germany: 1.52%, and US: 0.33%), all of which are strongly associated with increased rash severity in our findings. Many of those species are key fecal coliforms. In contrast, Chinese samples have the highest abundance of *Staphylococcus hominis* (China: 2.66%, Germany: 0.33%, and US: 0.57%) and *Bifidobacterium longum* (China: 27.14%, Germany: 19.33%, and US: 6.75%), which showed a negative impact on rash severity. These geographic differences align with our previous publication [1], suggesting that cultural practices and dietary habits can significantly influence the DDA outcomes. The higher microbial diversity observed in German samples may be attributed to residual fecal material and associated microbes. In contrast, the lower DNA yield and diversity in Chinese samples likely reflect more stringent cleaning practices by caregivers (Figure 4, Table S3). Additionally, Chinese infants tend to spend less time in overnight diapers [1], which may further contribute to these differences, linking to Chinese samples with the highest amount of commensal skin microbes such as *Moraxella osloensis* (China: 3.98%, Germany: 0.85%, and US: 0.27%) and *Corynebacterium amycolatum* (China: 1.38%, Germany: 0.01%, and US: 0.03%). These observations, alongside variations in skin pH and the prevalence of diaper dermatitis among populations in China, Germany, and the US, suggest that cultural, dietary, and environmental factors play significant roles.

These observations, alongside variations in skin pH and the prevalence of diaper dermatitis among populations in China, Germany, and US, suggest that cultural, dietary, and environmental factors play significant roles. Differences in diapering routines, skin care practices, and available diapering products may also drive these variations. Thus, it is essential to tailor infant care approaches to different cultural contexts, particularly regarding practices like air exposure to diapered skin and cleaning protocols, to better prevent perianal rashes. Ultimately, these insights may inform pediatric skin care guidelines aimed at reducing or preventing diaper dermatitis globally.

The findings of this study mostly align with and expand upon previous research regarding the role of specific microbial species in DDA. Consistent with earlier findings, our study confirms that *Bifidobacterium longum* is negatively correlated with rash severity, emphasizing its role in supporting the skin barrier and immune function. Furthermore, *Finegoldia* and *Staphylococcus* species has been previously linked to increased rash severity [14,16], our results showed that high levels of *Staphylococcus aureus* observed in perianal high rash samples and samples with elevated skin pH (Figure 5d), while *Finegoldia magna* only showed a significant correlation with buttock rash (Spearman correlation rho = 0.17, *p* value = 0.03), not a link to perianal rash or skin pH (Table S5). Additionally, the positive correlations of *Veillonella* species, particularly *Veillonella atypica* and *Enterococcus faecalis*, with perianal rash scores and pH resonate with earlier studies that reported the association of gut-associated bacteria or fecal coliform bacteria with DDA (Figure 5b,c). Another important fecal coliform *Escherichia coli* is also enriched (but not significant) in rash samples while associated positively with higher skin pH. Although *Candida albicans* is recognized as an important pathogen for DDA, we did not observe that in this population. In fact, *Candida albicans* was only detected in one sample out of these 158 infants’ 386 samples.

There are significant differences in microbial composition between buttocks and perianal samples, with gut-associated bacteria predominating in perianal samples. This shift in microbial profile has important implications for DDA severity. In perianal rash samples, we observed a higher abundance of gut microbes typically linked to digestive issues, while beneficial microbes that support immunity and gut barrier function were less abundant. The more severe rashes observed in the perianal area, compared to the buttocks, may result from fecal and urine accumulation, friction from skin-to-skin contact (intertrigo), as well as heat generation in this more occluded region, further contributing to the distinct microbial profiles. Notably, *Akkermansia* sp. *KLE1798*, a commensal, is found only in higher abundance in groups without rashes (Figure 5f). *Akkermansia* species are known for their mucin-degrading abilities, which nourish colon cells and support gut barrier integrity [32,33,34]. Certain *Akkermansia* species can metabolize Human Milk Oligosaccharides (HMOs) resulting in the production of organic acids which would lower or maintain skin pH in the healthy, acidic range, suggesting a role in infant health [35].

Alterations in skin pH can significantly influence microbial diversity and composition, potentially fostering an environment that promotes the development of diaper rash. There exists a bidirectional relationship between skin pH and microbial communities, suggesting that interventions aimed at maintaining lower pH levels may help mitigate the severity of DDA. Notably, in the buttock region, *Bifidobacterium longum* shows a negative correlation with both pH and rash severity (Figure 5e), indicating its potential as a target for probiotic-based interventions. Conversely, the perianal area appears to also be affected by factors such as osmolarity, heat, and degradative enzymes. Therefore, enhanced cleaning and protective measures in this region are crucial for preventing DDA.

In addition to microbial composition, we examined microbial biological pathways and gene functions associated with skin pH and diaper rash severity. Several pathways for amino acid, vitamin, and cofactor biosynthesis exhibited negative correlations with skin pH and DDA severity. This suggests that microbiota may play a role in supplying micronutrients that nourish the skin. On the other hand, the biosynthesis of L-ornithine and L-citrulline, along with polyamine production, is elevated in samples with high pH and high rash severity. Arginine, L-ornithine, L-citrulline, and polyamines (such as putrescine, spermidine, and spermine) are interconnected in metabolic pathways and serve critical roles in various physiological processes [36]. These metabolites are essential for wound healing, modulating immune responses, and regulating inflammation, all of which are crucial for skin health and recovery. Elevated levels of these microbial pathways in rash-affected areas may indicate an active metabolic response to tissue damage or inflammation, suggesting that the microbiota’s metabolic profile could significantly influence the skin’s ability to heal and maintain its barrier function. Pathways related to lipid and fatty acid biosynthesis were positively associated with perianal rash severity and skin pH but showed an inverse trend in buttocks rash (Figure 6). Given that lipids are essential for maintaining skin barrier function and integrity, and fatty acids can influence skin pH, these data suggest that microbial metabolites may play a key role in regulating the skin environment. Subsequently, dysregulated metabolic processes may contribute to the pathophysiology of DDA. Several cell surface virulence factors, such as lipid A and O-antigen biosynthesis (both associated with Gram-negative bacteria), correlated positively with DDA severity, while peptidoglycan biosynthesis (linked to Gram-positive bacteria) correlated negatively. This aligns with our earlier finding that higher levels of fecal coliforms, predominantly Gram-negative bacteria, on infant skin may correlate with an increased risk of DDA.

Moreover, many stress response genes were elevated in rash samples, particularly those related to oxidative response and osmolarity, while responses to heat showed a negative correlation with both pH and rash severity (Figure 7). A potential link exists between osmolarity stress and homeostatic transport functions, which were elevated in cases of higher skin pH and perianal rash but did not correlate strongly with buttocks rash. This supports our hypothesis that perianal rashes may be more significantly affected by residual urine and fecal matter, leading to increased stress for microbes and irritation for the infant’s skin. As osmolarity and oxidative stress rise, microbes may activate detoxification pathways or biosynthesize substances like rhamnose to manage these stressors.

This manuscript presents a comprehensive analysis that integrates the contributions of skin pH and site-specific microbiome compositions (buttocks and perianal) across diverse infant populations to investigate the causes of DDA, a prevalent health concern for caregivers that remains underexplored, as evidenced by its high prevalence across all three geographies studied. By identifying specific microbial species correlated with DDA severity and skin pH, our study provides valuable insights into the microbial dynamics associated with DDA, paving the way for potential probiotic or microbiome-targeted interventions. Furthermore, these data suggest a necessity for the development of diapering interventions which support a healthy microbiome in the diapered area to mitigate the dysbiosis observed in DDA. The inclusion of infants from multiple countries allows for an examination of geographical variability in skin pH and microbiome composition, enhancing our understanding of how cultural and environmental factors impact infant skin health. The current findings across all three geographies underscore the importance of maintaining a slightly acidic skin pH on both non-diapered and diapered areas to reduce the incidence and severity of DDA. Caregivers should be aware of how products and behaviors can influence skin pH. When feasible, especially for patients with frequent or severe DDA, integrating skin pH monitoring into routine pediatric care is recommended. Our analysis of metabolic pathways and gene functions related to skin pH and diaper rash severity further enriches the understanding of the biological mechanisms underlying DDA. Understanding the factors influencing DDA has broader public health implications for infant care practices and recommendations. These findings can guide caregivers and healthcare professionals in implementing effective strategies to prevent and manage DDA, ultimately improving infant health outcomes. Insights from this study will also inform future research directions, paving the way for further exploration of the intricate dynamics between skin pH, microbiome health, and DDA.

The study is not without limitations, including the lack of longitudinal data, potential limitation for the classification of microbes through current available libraries, the use of tape strips for microbiome sampling (which may capture more background microbes than swabs), and the use of buttock pH due to inability to obtain perianal pH. Future research should focus on longitudinal studies to track changes in skin measurements, such as pH stratum corneum hydration, trans-epidermal water loss (TEWL), and microbiome composition related to DDA development over time, particularly addressing microbial functional changes to enhance understanding of effective prevention strategies.

## 5. Conclusions

In conclusion, our study underscores the multifaceted relationship between skin pH, microbiome composition, and diaper rash severity in infants. Understanding the roles of specific microbial species and metabolic pathways will be essential for developing effective strategies to prevent and manage DDA, and the current data point to skin pH as a potential key regulator of microbiome diversity. Future research should focus on longitudinal studies to explore how these factors evolve over time and how they may inform personalized care approaches in pediatric dermatology.

## Data Availability

Data and R code will be available upon request. Genomics sequences from the 386 samples were deposited into the NCBI SRA database with Bioproject PRJNA1346863.

## Supplementary Materials

The following supporting information can be downloaded at: https://www.mdpi.com/article/10.3390/microorganisms13112632/s1, Figure S1: Microbial Pathway Heatmap of 182 microbial pathways showing Spearman correlations with skin pH or rash scores (*p* ≤ 0.05) in perianal or buttocks sites; Figure S2: Selected Microbial Gene GO Biological Process Terms Heatmap showing Spearman correlations with skin pH or rash scores (*p* ≤ 0.05) in perianal or buttocks sites; Figure S3: Microbial Diversity and Their Associations with Rash and pH in Diapered Areas (Perianal and Buttocks); Table S1: Total DNA amount (ng) extracted from Tape strips of Infant skins by anatomical sites and geographic locations; Table S2: Detected Total Microbial Species Number from Tape strips of Infant skins by anatomical sites and geographic locations; Table S3: Top Infant Skin Microbial Species (>1% of Relative Abundance ) by Geographic Location; Table S4: Top Infant Skin Microbial Species (>1% of Relative Abundance or top fungal species) by anatomical site; Table S5: Correlation of Top Buttocks Bacterial Species (with Relative Abundance higher than 1%) to Buttocks Rash Severity; Table S6: Correlation of Top Perianal Bacterial Species (with Relative Abundance higher than 1%) to Perianal Rash Severity; Table S7: Correlation of representative Buttocks Microbial Species (with Relative Abundance higher than 1% or selected pathogens) to Buttocks Skin pH; Table S8: Correlation of top Perianal Bacterial Species (with Relative Abundance higher than 1%) to Buttocks Skin pH; Table S9: Gender Distribution of the Subjects Involved in this analysis; Table S10: Correlation analysis of gender to skin pH or rash severity of the 158 infants in this study showed no significant correlation among gender to skin pH or rash severity.
