# Supplementary material for: Metagenomics Investigation on Baby Diaper Area Microbiome and Its Association with Skin pH and Dermatitis in the Diapered Area
Source: Microorganisms. 2025 Nov 20;13(11):2632. doi: 10.3390/microorganisms13112632 (PMC12654720; doi:10.3390/microorganisms13112632)
Supplement: Supplementary file 1 [file microorganisms-13-02632-s001.zip › microorganisms-3926385-supplementary.pdf]

microorganisms

MDPI

+*Article*

Metagenomics Investigation on Baby Diaper Area Microbiome and Its Association with Skin pH and Dermatitis in the Diapered Area

Ping Hu [1], Andrew N. Carr [2], Mirjana Parlov[1], Dionne Swift [1], Jay P. Tiesman[1], Nivedita Ramji[1], Jennifer J. Schoch[3], and Amber G. Teufel [2,*]

Supplementary Materials:

Table S1. Total DNA amount (ng) extracted from Tape stripes of Infant skins by anatomical sites and geographic locations

Group	Measurement	group1	Mean1	group2	Mean2	n1	n2	*p*	*p*.adj
**All geographic location**	DNAng	Buttocks	7.23	Perianal	15.24	158	158	**1.62E-06**	3.24E-06
**All geographic location**	DNAng	Buttocks	7.23	Thigh	6.63	158	158	**2.73E-05**	2.73E-05
**All geographic location**	DNAng	Perianal	15.24	Thigh	6.63	158	158	**2.35E-07**	7.05E-07
**All anatomical site**	DNAng	CHINA	7.16	GERMANY	9.78	96	186	**0.0881**	0.176
**All anatomical site**	DNAng	CHINA	7.16	USA	10.90	96	192	**0.0146**	0.0439
**All anatomical site**	DNAng	GERMANY	9.78	USA	10.90	186	192	**0.374**	0.374
**Buttocks**	DNAng	CHINA	6.98	GERMANY	7.14	32	62	**0.744**	1
**Buttocks**	DNAng	CHINA	6.98	USA	7.45	32	64	**0.345**	1
**Buttocks**	DNAng	GERMANY	7.14	USA	7.45	62	64	**0.453**	1
**Perianal**	DNAng	CHINA	8.02	GERMANY	15.69	32	62	**0.0752**	0.15
**Perianal**	DNAng	CHINA	8.02	USA	18.41	32	64	**0.0159**	0.0476
**Perianal**	DNAng	GERMANY	15.69	USA	18.41	62	64	**0.439**	0.439
**Thigh**	DNAng	CHINA	6.49	GERMANY	6.51	32	62	**0.959**	0.959
**Thigh**	DNAng	CHINA	6.49	USA	6.83	32	64	**0.282**	0.642
**Thigh**	DNAng	GERMANY	6.51	USA	6.83	62	64	**0.214**	0.642
**CHINA**	DNAng	Buttocks	6.98	Perianal	8.02	32	32	**0.00391**	0.00782
**CHINA**	DNAng	Buttocks	6.98	Thigh	6.49	32	32	**0.164**	0.164
**CHINA**	DNAng	Perianal	8.02	Thigh	6.49	32	32	**3.31E-05**	9.93E-05
**GERMANY**	DNAng	Buttocks	7.14	Perianal	15.69	62	62	**0.000183**	0.000367
**GERMANY**	DNAng	Buttocks	7.14	Thigh	6.51	62	62	**0.776**	0.776
**GERMANY**	DNAng	Perianal	15.69	Thigh	6.51	62	62	**6.12E-05**	0.000184
**USA**	DNAng	Buttocks	7.45	Perianal	18.41	64	64	**4.46E-06**	8.93E-06
**USA**	DNAng	Buttocks	7.45	Thigh	6.83	64	64	**0.79**	0.79
**USA**	DNAng	Perianal	18.41	Thigh	6.83	64	64	**1.35E-06**	4.04E-06

Table S2. Detected Total Microbial Species Number from Tape stripes of Infant skins by anatomical sites and geographic locations

Group	Measurement	group1	Mean 1	group2	Mean 2	n1	n2	*p*	*p*.adj
**All geographic location**	SpeciesNum	Thigh	73.41	Buttocks	58.94	70	158	0.00555	0.0111

**All geographic location**	SpeciesNum	Thigh	73.41	Perianal	55.15	70	158	0.000482	0.00145
**All geographic location**	SpeciesNum	Buttocks	58.94	Perianal	55.15	158	158	0.351	0.351
**All anatomical site**	SpeciesNum	CHINA	38.91	USA	74.12	74	164	1.14E-12	3.43E-12
**All anatomical site**	SpeciesNum	CHINA	38.91	GERMANY	54.93	74	148	0.00108	0.00108
**All anatomical site**	SpeciesNum	USA	74.12	GERMANY	54.93	164	148	1.1E-06	2.2E-06
**Thigh**	SpeciesNum	CHINA	41.70	USA	86.53	10	36	0.00681	0.0204
**Thigh**	SpeciesNum	CHINA	41.70	GERMANY	66.96	10	24	0.14	0.206
**Thigh**	SpeciesNum	USA	86.53	GERMANY	66.96	36	24	0.103	0.206
**Buttocks**	SpeciesNum	CHINA	36.69	USA	74.78	32	64	7.53E-07	2.26E-06
**Buttocks**	SpeciesNum	CHINA	36.69	GERMANY	54.08	32	62	0.0204	0.0204
**Buttocks**	SpeciesNum	USA	74.78	GERMANY	54.08	64	62	0.000841	0.00168
**Perianal**	SpeciesNum	CHINA	40.25	USA	66.48	32	64	2.05E-05	6.14E-05
**Perianal**	SpeciesNum	CHINA	40.25	GERMANY	51.13	32	62	0.0718	0.0718
**Perianal**	SpeciesNum	USA	66.48	GERMANY	51.13	64	62	0.00212	0.00424
**CHINA**	SpeciesNum	Thigh	41.70	Buttocks	36.69	10	32	0.446	1
**CHINA**	SpeciesNum	Thigh	41.70	Perianal	40.25	10	32	0.825	1
**CHINA**	SpeciesNum	Buttocks	36.69	Perianal	40.25	32	32	0.432	1
**USA**	SpeciesNum	Thigh	86.53	Buttocks	74.78	36	64	0.194	0.388
**USA**	SpeciesNum	Thigh	86.53	Perianal	66.48	36	64	0.0274	0.0823
**USA**	SpeciesNum	Buttocks	74.78	Perianal	66.48	64	64	0.279	0.388
**GERMANY**	SpeciesNum	Thigh	66.96	Buttocks	54.08	24	62	0.0531	0.106
**GERMANY**	SpeciesNum	Thigh	66.96	Perianal	51.13	24	62	0.0178	0.0534
**GERMANY**	SpeciesNum	Buttocks	54.08	Perianal	51.13	62	62	0.551	0.551

Table S3. Top Infant Skin Microbial Species (>1% of Relative Abundance) by Geographic Location

**Species**	**Relative Abundance %**			***p* Kruskal**	***p* Wilcoxon**		
Taxon Name	CHINA	US	GERMANY	Location	CHINA: GERMANY	CHINA: US	GERMANY: US
Bifidobacterium longum	27.14	6.35	19.33	1.38E-07	6.68E-01	3.81E-03	8.97E-09
Bifidobacterium breve	7.52	5.11	4.51	3.05E-02	9.18E-02	8.69E-01	1.07E-02
Bifidobacterium pseudocatenulatum	4.54	1.57	1.39	1.16E-03	1.18E-01	1.85E-01	2.22E-04
Moraxella osloensis	3.98	0.27	0.85	8.50E-10	9.70E-06	1.55E-10	2.37E-02
Staphylococcus haemolyticus	3.42	0.14	0.28	2.42E-24	1.83E-19	3.35E-18	4.19E-01
Bifidobacterium bifidum	3.35	2.25	3.23	3.69E-03	3.98E-02	7.69E-01	1.14E-03
Veillonella parvula	1.66	1.64	3.24	1.59E-03	1.10E-01	1.15E-01	4.41E-04
Serratia liquefaciens	2.37	3.21	2.35	1.34E-01	3.77E-02	2.45E-01	3.52E-01
Finegoldia magna	3.19	2.85	1.47	1.08E-04	2.45E-01	5.83E-02	1.38E-05
Staphylococcus hominis	2.66	0.57	0.33	9.75E-04	2.08E-04	7.08E-03	2.26E-01
Escherichia coli	0.36	0.58	2.57	2.75E-09	4.06E-07	2.16E-01	8.53E-08
Cutibacterium acnes	0.57	2.38	1.33	3.66E-01	1.80E-01	7.35E-01	2.88E-01
Ruminococcus gnavus	0.98	2.29	1.95	2.30E-13	8.85E-08	2.50E-14	3.25E-03
Phocaeicola vulgatus	0.11	1.76	2.14	2.05E-09	4.50E-04	2.03E-10	1.96E-03

Faecalibacterium prausnitzii	0.00	2.10	1.51	6.17E-12	2.47E-05	1.74E-11	6.17E-05
Streptococcus mitis	0.48	1.89	1.38	5.91E-02	2.08E-02	4.07E-02	8.41E-01
Prevotella bivia	1.89	0.93	1.88	3.54E-01	2.92E-01	9.09E-01	1.92E-01
Lawsonella SGB3665	0.78	1.83	1.67	3.50E-03	8.15E-02	1.04E-03	5.97E-02
Bacteroides fragilis	0.04	0.63	1.79	3.66E-03	9.65E-04	6.45E-03	2.70E-01
Segatella copri	0.00	1.79	1.11	4.66E-06	2.27E-02	3.60E-05	1.03E-03
Anaerococcus obesiensis	1.06	1.71	0.88	2.43E-04	5.87E-02	2.15E-04	6.30E-03
Pseudomonas fluorescens	1.44	1.59	1.44	1.48E-01	4.75E-02	1.46E-01	5.81E-01
Enterococcus faecalis	0.65	0.98	1.55	2.91E-05	1.11E-03	9.00E-01	2.47E-05
Prevotella timonensis	1.22	1.53	1.22	4.09E-03	2.48E-01	2.82E-03	2.05E-02
Veillonella atypica	0.29	0.33	1.52	3.67E-06	1.73E-03	4.91E-01	1.69E-06
Klebsiella pneumoniae	1.48	0.09	0.06	3.51E-02	1.19E-02	5.15E-02	4.57E-01
Corynebacterium amycolatum	1.38	0.03	0.01	1.14E-03	2.65E-04	2.07E-02	9.03E-02
Varibaculum cambriense	1.34	0.46	0.32	1.72E-02	5.11E-01	1.48E-02	2.62E-02
Veillonella ratti	0.09	1.34	0.12	1.15E-09	8.12E-02	6.28E-04	4.88E-09
Staphylococcus epidermidis	1.32	1.10	0.74	6.38E-01	3.49E-01	5.30E-01	6.72E-01
Akkermansia muciniphila	0.07	1.30	0.59	3.22E-04	4.46E-02	2.45E-04	1.40E-02
Peptoniphilus harei	1.19	1.12	0.53	1.80E-03	7.01E-01	3.88E-02	5.11E-04
Streptococcus salivarius	1.19	0.77	0.55	3.99E-03	4.79E-03	3.54E-01	6.21E-03
Blautia wexlerae	0.08	1.17	0.67	1.99E-11	5.05E-04	1.25E-10	1.99E-05
Prevotella buccalis	0.25	1.12	0.71	1.55E-05	5.23E-02	1.64E-05	1.98E-03
Veillonella seminalis	0.00	0.18	1.07	3.34E-07	1.35E-04	1.31E-01	2.25E-05
Levyella massiliensis	0.51	1.06	0.22	4.86E-04	9.86E-01	1.01E-02	3.72E-04
Erysipelatoclostridium ramosum	0.04	1.03	0.54	2.49E-10	2.43E-07	3.39E-11	2.76E-02

Table S4. Top Infant Skin Microbial Species (>1% of Relative Abundance or top fungal species) by anatomical site

**Species**	**Relative Abundance %**			***p* Kruskal**		***p* Wilcoxon**	
**TaxonName**	Buttocks	Perianal	Thigh	Site	Buttocks: Perianal	Buttocks: Thigh	Perianal:Thigh
Bifidobacterium longum	21.414	12.353	8.220	3.46E-04	2.23E-03	3.99E-04	2.32E-01
Serratia liquefaciens	2.858	0.315	7.838	1.53E-45	1.31E-29	1.82E-13	7.99E-30
Cutibacterium acnes	0.648	0.041	7.437	1.68E-52	6.15E-31	7.29E-24	1.86E-36
Streptococcus mitis	0.432	0.052	6.759	1.11E-38	2.22E-11	9.55E-25	4.75E-32
Bifidobacterium breve	7.002	4.108	4.367	1.85E-01	1.19E-01	1.39E-01	7.45E-01
Pseudomonas fluorescens	1.745	0.191	3.939	2.51E-44	1.66E-29	1.14E-12	1.20E-28
Moraxella osloensis	1.083	0.253	3.617	3.71E-23	2.47E-12	1.52E-07	2.56E-22
Streptococcus salivarius	0.514	0.319	2.358	8.78E-10	1.07E-04	4.49E-05	4.11E-09
Rothia mucilaginosa	0.109	0.025	2.129	4.69E-26	2.44E-02	1.91E-18	2.05E-23
Streptococcus lactarius	0.090	0.055	1.954	5.78E-06	1.11E-01	2.12E-04	2.39E-06
Staphylococcus epidermidis	1.441	0.240	1.747	3.29E-20	2.39E-05	1.33E-10	1.53E-20
Staphylococcus hominis	1.137	0.276	1.656	1.06E-12	1.63E-04	2.96E-06	3.34E-13
Micrococcus luteus	0.214	0.027	1.550	3.13E-24	2.73E-12	8.34E-09	7.97E-24
Bifidobacterium bifidum	4.128	2.183	1.411	4.16E-02	6.85E-02	2.62E-02	2.67E-01
Segatella copri	1.515	0.824	1.275	1.19E-01	6.73E-01	1.11E-01	4.81E-02
Ruminococcus gnavus	2.780	1.369	1.162	9.65E-02	2.54E-01	3.82E-02	1.77E-01

Finegoldia magna	1.054	4.294	1.067	1.62E-12	1.50E-11	6.98E-01	9.91E-08
Streptococcus peroris	0.066	0.021	1.024	2.59E-14	1.13E-03	4.17E-08	4.00E-14
Bifidobacterium pseudocatenulatum	3.081	1.547	0.964	6.77E-01	4.80E-01	4.53E-01	8.38E-01
Anaerococcus obesiensis	0.593	2.292	0.476	4.08E-04	9.08E-04	4.00E-01	1.40E-03
Phocaeicola vulgatus	1.999	1.690	0.430	6.72E-01	7.29E-01	3.87E-01	5.16E-01
Faecalibacterium prausnitzii	1.840	1.576	0.407	7.49E-01	6.27E-01	4.67E-01	7.40E-01
Veillonella parvula	1.673	3.664	0.404	1.09E-03	1.00E-02	1.57E-01	6.13E-04
Veillonella atypica	0.412	1.317	0.400	1.19E-01	4.38E-02	2.99E-01	4.63E-01
Enterococcus faecalis	1.047	1.553	0.381	1.01E-04	1.12E-01	1.43E-03	2.38E-05
Prevotella timonensis	0.625	2.533	0.323	7.07E-03	3.40E-03	7.41E-01	3.44E-02
Blautia wexlerae	1.238	0.503	0.312	6.19E-01	3.32E-01	8.80E-01	5.70E-01
Staphylococcus haemolyticus	1.312	0.572	0.294	4.01E-01	1.82E-01	7.23E-01	4.70E-01
Prevotella corporis	0.287	1.269	0.273	3.91E-01	1.84E-01	7.93E-01	4.37E-01
Lawsonella SGB3665	0.382	3.331	0.267	1.29E-13	1.09E-11	5.61E-01	7.28E-09
Peptoniphilus harei	0.297	1.806	0.266	1.59E-07	1.24E-06	3.73E-01	1.48E-05
Escherichia coli	1.759	1.301	0.256	1.92E-08	1.69E-01	1.60E-06	2.34E-09
Bacteroides fragilis	0.989	1.266	0.218	5.61E-01	6.10E-01	4.68E-01	3.01E-01
Prevotella bivia	0.482	3.028	0.215	3.63E-01	2.20E-01	8.76E-01	2.62E-01
Prevotella buccalis	0.191	1.672	0.169	2.94E-07	1.94E-05	7.70E-02	6.71E-06
Varibaculum cambriense	0.259	1.082	0.143	1.27E-07	3.42E-06	1.93E-01	6.78E-06
Prevotella disiens	0.259	1.323	0.095	1.14E-01	2.18E-01	2.66E-01	4.65E-02
Malassezia restricta	0.001	0.000	0.098	3.47E-27	2.46E-02	4.73E-15	1.64E-18
Malassezia globosa	0.027	0.000	0.040	1.72E-02	7.61E-03	6.67E-01	2.53E-03
Candida albicans	0.001	0.000	0.000	4.86E-01	3.20E-01	5.12E-01	1.00E+00

Table S5. Correlation of Top Buttocks Bacterial Species (with Relative Abundance higher than 1%) to Buttocks Rash Severity

**Buttocks Samples: Rash**	**Relative Abundance**			**Spearman Correlation**		**Wilcoxon *P* value**		
**Species**	No Rash (119)	Mild Rash (34)	High Rash (5)	Cor Rash	*P* Rash	No Rash: Mild Rash	No Rash: High Rash	Mild Rash: High Rash
Bifidobacterium longum	26.47	6.19	4.72	-0.29	0.0002	0.0008	0.1288	0.9832
Bifidobacterium catenulatum	0.74	0.66	8.64	0.16	0.0440	0.3300	0.0016	0.0231
Staphylococcus epidermidis	0.72	4.08	0.58	0.18	0.0205	0.0472	0.3232	1.0000
Finegoldia magna	1.04	1.19	0.42	0.17	0.0337	0.0345	0.9287	0.3887

Table S6. Correlation of Top Perianal Bacterial Species (with Relative Abundance higher than 1%) to Perianal Rash Severity

Perianal Samples: Perianal Rash	Relative Abundance			Spearman Correlation		Wilcoxon ***P*** value		
Species	No Rash (30)	Mild Rash (67)	High Rash (61)	Cor Rash	*P* Rash	No Rash: Mild Rash	No Rash: High Rash	Mild Rash: High Rash
Veillonella atypica	0.54	**0.40**	**2.71**	0.35	0.0000	0.8819	0.0003	0.0000
Veillonella parvula	**1.92**	3.68	**4.51**	0.24	0.0023	0.0414	0.0104	0.3751
Enterococcus faecalis	**0.53**	1.47	**2.15**	0.24	0.0024	0.0447	0.0041	0.1963

Phocaeicola dorei	**0.01**	0.37	**1.90**	0.23	0.0036	0.8278	0.0284	0.0069
Veillonella seminalis	**0.00**	0.70	**1.67**	0.20	0.0128	0.1291	0.0201	0.1395
GGB4277 SGB5832	**1.25**	**0.81**	1.02	-0.18	0.0230	0.0200	0.0101	0.7947
Faecalibacterium prausnitzii	**3.10**	**1.00**	1.46	-0.19	0.0182	0.0003	0.0054	0.3739
Segatella copri	**1.33**	**0.35**	1.10	-0.19	0.0179	0.0005	0.0144	0.3384
Phocaeicola vulgatus	**2.68**	**1.31**	1.62	-0.26	0.0009	0.0008	0.0007	0.9505
Porphyromonas sp HMSC065F10	**3.70**	0.43	**0.08**	-0.27	0.0005	0.0158	0.0004	0.1139
Veillonella ratti	**1.25**	**0.51**	1.26	-0.28	0.0004	0.0082	0.0006	0.2817
Bacteroides caccae	**2.11**	**0.07**	0.13	-0.29	0.0002	0.0000	0.0001	0.9932

Table S7. Correlation of representative Buttocks Microbial Species (with Relative Abundance higher than 1% or selected pathogens) to Buttocks Skin pH.

**Buttocks Species: Buttocks pH**	**Relative Abundance**		**Spearman Correlation**		**Buttocks: LowpH vs. HighpH**	
**Species**	High PH (72)	Low pH (86)	Cor	P Value	P value Wilcoxon	Fold
Escherichia coli	3.2257	0.5320	0.19	0.02	0.0482	-6.06
Moraxella osloensis	1.8752	0.4192	0.16	0.05	0.1098	-4.47
Staphylococcus aureus	0.0645	0.0152	0.16	0.05	0.0210	-4.24
Malassezia restricta	0.0010	0.0003	0.18	0.03	0.1215	-3.14

Table S8. Correlation of top Perianal Bacterial Species (with Relative Abundance higher than 1%) to Buttocks Skin pH.

**Perianal Species: Buttocks pH**	**Relative Abundance**		**Spearman Correlation**		**Perianal: LowpH vs. HighpH**	
**Species**	High PH	Low pH	Cor	P Value	P value Wilcoxon	Fold
Prevotella bivia	4.11	2.13	0.20	0.01	0.0737	-1.93
Escherichia coli	1.85	0.84	0.19	0.02	0.1349	-2.20
Segatella copri	1.46	0.29	0.17	0.04	0.1209	-5.08
Veillonella seminalis	1.06	0.84	0.19	0.02	0.0254	-1.26

Table S9. Gender Distribution of the Subjects Involved in this analysis

**Subject Number**	**CHINA**	**GERMANY**	**USA**	**Grand Total**
FEMALE	13	36	33	82
MALE	19	26	31	76
Grand Total	32	62	64	158

Table S10. Correlation analysis of gender to skin pH or rash severity of the 158 infants in this study showed no significant correlation among gender to skin pH or rash severity

Comparison		Pearson Correlation		Spearman Correlation	
Variable 1	Variable 2	rho	*p* value	rho	*p* value
Total Rash	GenderF1M2	0.04	0.58	-0.12	0.15
Buttocks Rash	GenderF1M2	0.03	0.67	0.03	0.73
Perianal Rash	GenderF1M2	0.04	0.62	-0.05	0.57
Buttocks pH	GenderF1M2	-0.15	0.06	-0.14	0.07
Genitals pH	GenderF1M2	0.05	0.53	0.08	0.33
Thigh pH	GenderF1M2	0.03	0.70	0.02	0.78

Figure S1. Microbial Pathway Heatmap of 182 microbial pathways showing Spearman correlations with skin pH or rash scores (p ⩽ 0.05) in perianal or buttocks sites. Side color bar indicated functional groups of these pathways. Panel 1 shows Spearman correlation coefficients with pH or rash scores (p ⩽ 0.05) in perianal or buttocks sites. Panel 2 shows Z-score﹣normalized relative abundance per species across groups. Panel 3 shows fold changes with *p*-values from pairwise Wilcoxon tests (p ⩽ 0.05: *, FDR

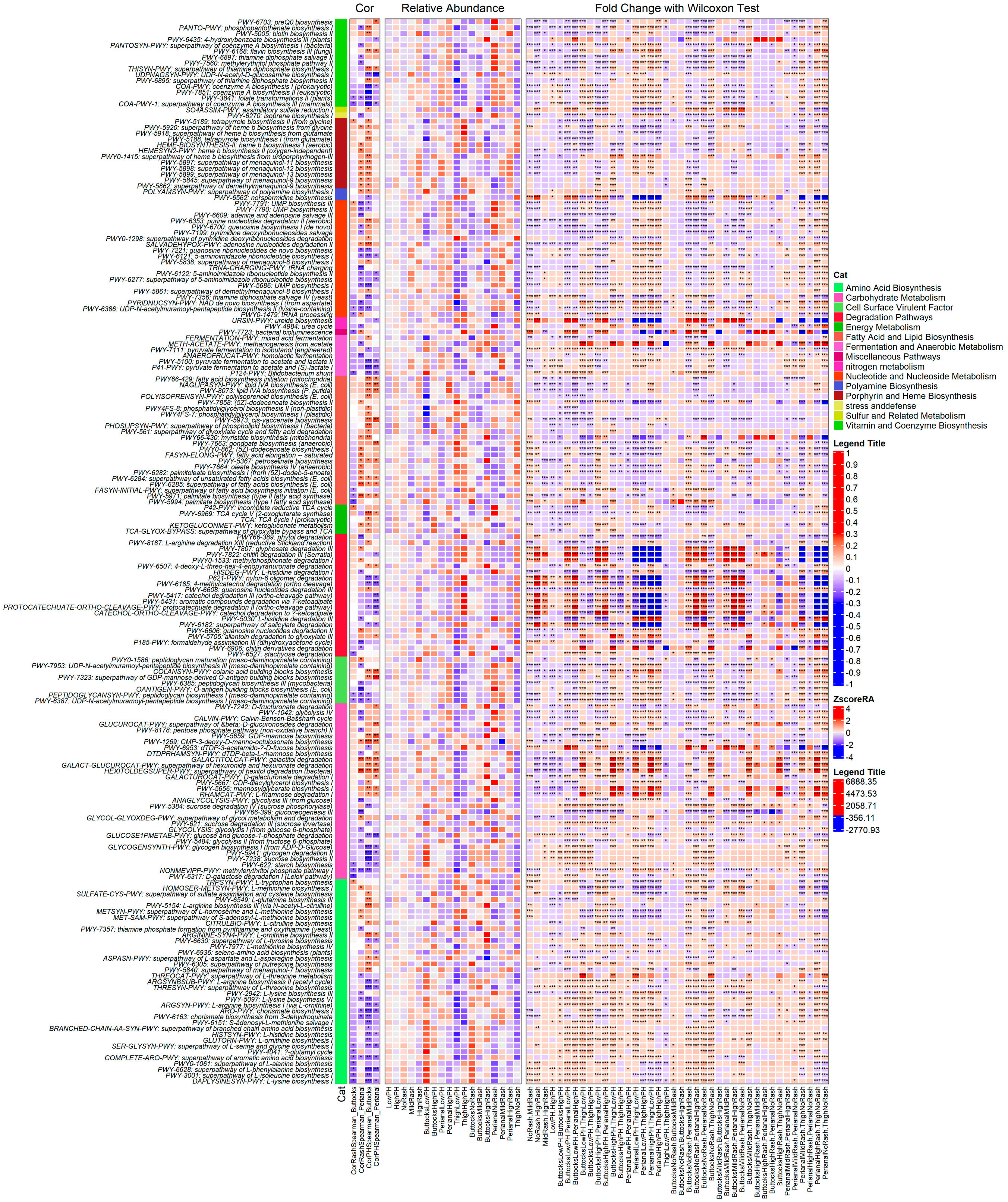

Figure S2. Selected Microbial Gene GO Biological Process Terms Heatmap showing Spearman correlations with skin pH or rash scores (p ≤ 0.05) in perianal or buttocks sites. Side color bar indicated functional groups of these GO terms. Panel 1 shows Spearman correlation coefficients with pH or rash scores (p ≤ 0.05) in perianal or buttocks sites. Panel 2 shows Z-score - normalized relative abundance per species across groups. Panel 3 shows fold changes with *p*-values from pairwise Wilcoxon tests (p ≤ 0.05: *, FDR ≤ 0.1: **, FDR ≤ 0.05: ***).

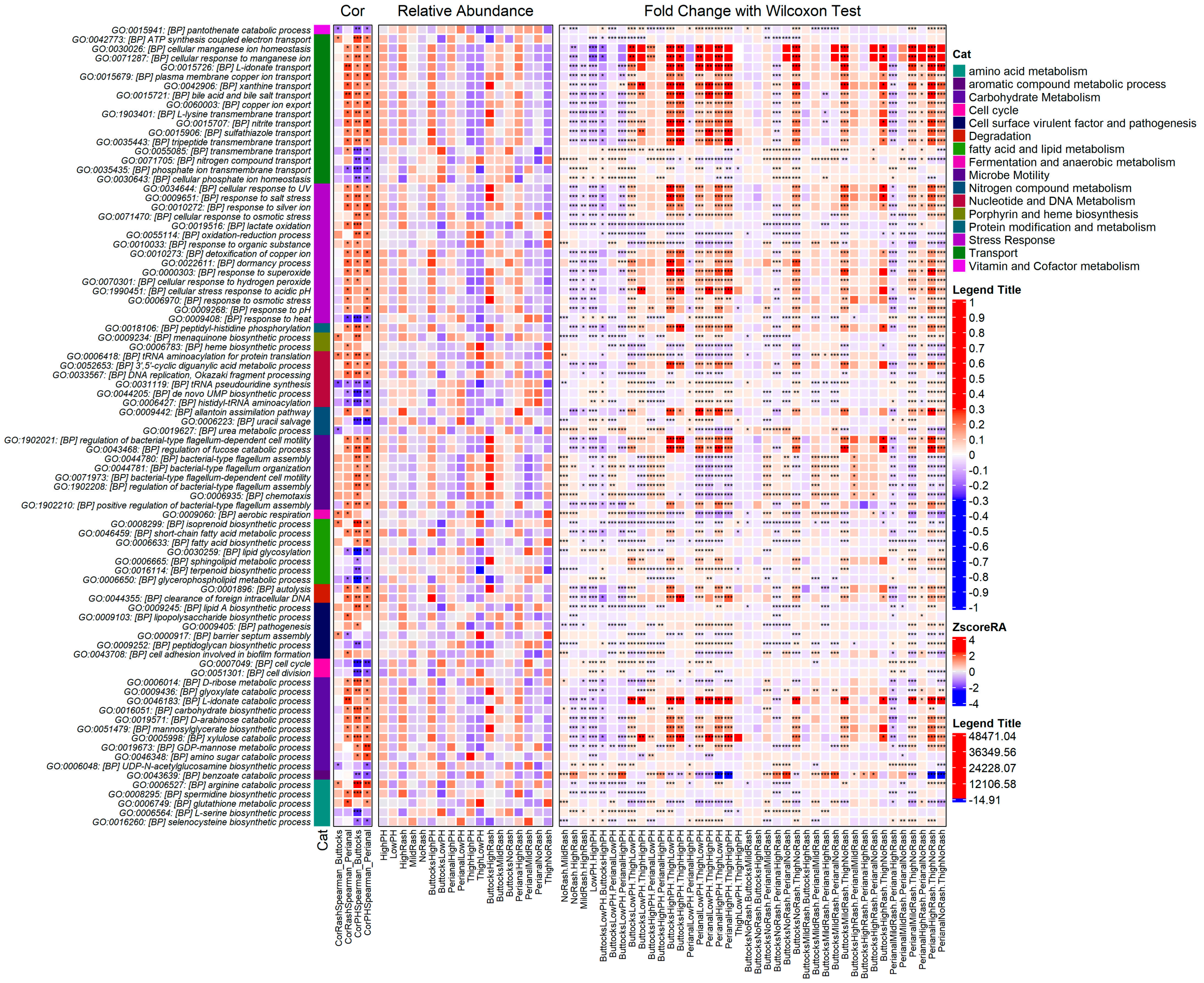

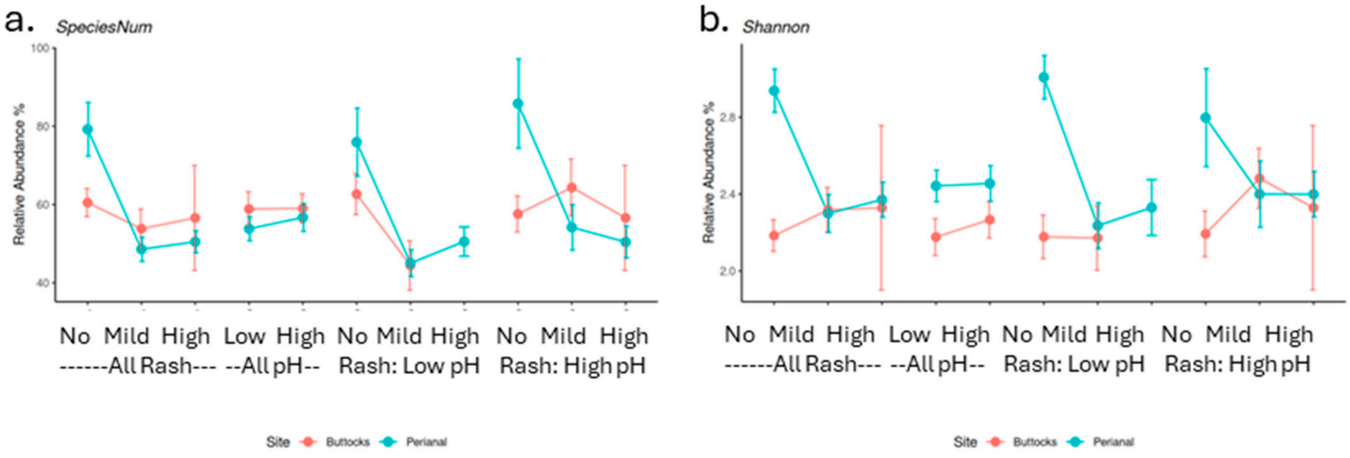

Figure S3. Microbial Diversity and Their Associations with Rash and pH in Diapered Areas (Perianal and Buttocks). The blue line indicates the relative abundance in perianal samples, while the red line represents the relative abundance in buttocks samples.
